# The safety and effectiveness of sintilimab versus camrelizumab, both plus targeted drugs, in advanced hepatocellular carcinoma

**DOI:** 10.3389/fimmu.2025.1585956

**Published:** 2025-06-23

**Authors:** Chenglong Zhao, Qiongni Zhu, Xiaoyan Qian, Zhonghua Fu, Wei Zhang, Yaqin Wang

**Affiliations:** ^1^ Department of Pharmacy, Henan Provincial People’s Hospital, People’s Hospital of Zhengzhou University, School of Clinical Medicine, Henan University, Zhengzhou, Henan, China; ^2^ Department of Pharmacy, Ruijin Hospital, Shanghai Jiao Tong University School of Medicine, Shanghai, China; ^3^ Department of Oncology, Henan Provincial People’s Hospital, People’s Hospital of Zhengzhou University, People’s Hospital of Henan University, Zhengzhou, Henan, China

**Keywords:** sintilimab, camrelizumab, hepatocellular carcinoma, survival analysis, real-world study

## Abstract

**Objectives:**

We aimed to evaluate the effectiveness and safety of sintilimab versus camrelizumab, both plus targeted drugs, for advanced hepatocellular carcinoma (HCC) in the real world. Then the effectiveness was compared between sintilimab-lenvatinib and camrelizumab-apatinib.

**Methods:**

Patients diagnosed as advanced HCC were included from January 2017 to December 2023. They were concurrently treated with targeted drugs and sintilimab or camrelizumab. Progression-free survival (PFS), overall survival (OS), and adverse events (AEs) were assessed. The subgroups analysis of PFS and OS based on baseline characteristics and univariate and multivariate COX analysis were done to test for heterogeneity of data and correct for confounding factors. Then subgroup analysis of sintilimab-lenvatinib versus camrelizumab-apatinib was conducted.

**Results:**

A total of 300 eligible HCC patients (199 in sintilimab group and 101 in camrelizumab group) were included in our study. No significant difference in PFS (p=0.47) and OS (p=0.51) was observed between sintilimab and camrelizumab groups. The median PFS (mPFS) was 262 days in sintilimab group, and 220 days in camrelizumab group, and neither group has reached the median OS. There was no difference in AEs between two groups also. The effect of sintilimab and camrelizumab on PFS and OS based on baseline characteristics was consistent with primary outcomes, except for other metastatic sites and lenvatinib in terms of OS. Multivariable Cox analysis identified the number of metastatic sites ≥2 and AFP level ≥400ng/mL as independent predictors of shorter PFS and OS, but they had no effect on the primary outcomes. In subgroup analysis, the PFS and OS of sintilimab -lenvatinib and camrelizumab-apatinib in first-line treatment of advanced HCC were not clinically different, although sintilimab-lenvatinib had a longer mPFS (301 days in sintilimab-lenvatinib group vs. 194 days in camrelizumab-apatinib group).

**Conclusions:**

Sintilimab and camrelizumab, both plus targeted agents, have equal clinical effectiveness and incidences of AEs. The effectiveness of sintilimab-lenvatinib and camrelizumab-apatinib are similar in first-line treatment of advanced HCC, despite a slight superiority in sintilimab-lenvatinib is observed.

## 1 Introduction

Hepatocellular carcinoma (HCC) is one of the most common malignant tumors in the world, with high morbidity and mortality. According to reports, 49.3% of new cases of liver cancer and 58.3% of deaths occurred in China (1). HCC has become the fourth malignant tumor with the fourth incidence rate and the second mortality rate in China (2). Due to the insidious onset of HCC, 70-80% of patients are initially diagnosed at a middle or advanced stage and miss opportunity for surgery (3). HCC with distant metastasis or progression has a dismal prognosis, with a 5-year survival rate of merely 3.1% for metastatic HCC (4).

Currently, systemic therapy is the mainstay of treatment for patients with unresectable or recurrent HCC, with the aim of controlling disease progression as much as possible and prolonging the survival time of patients (5). In 2007, the targeted antiangiogenic agent, sorafenib, was approved for the treatment of HCC (6), and then a variety of targeted agents appeared, such as bevacizumab (7), lenvatinib (8), apatinib (9), cabozantinib (10), et al. Given that targeted agents can no longer meet the clinical needs for lacking of alternative drugs after targeted therapy failure, researchers are exploring new therapeutic options to prolong the survival of patients with advanced HCC, such as immunotherapy combined with targeted therapy.

Two reviews revealed that immunotherapeutic drugs and antiangiogenic drugs theoretically have synergistic effects. Angiogenesis is an important step in the process of tumor growth and metastasis. Neoplastic tumor blood vessels, due to abnormal perfusion and increased permeability, cause tissue hypoxia and promote the release of tissue factor, which not only inhibits the function of immune cells in the tumor microenvironment, but also produces systemic immunosuppressive effects through blood circulation. Anti-angiogenic drugs can restore the balance in terms of angiogenesis in the tumor, remodel the tumour microenvironment (TME), promote T cell infiltration into the tumor, and then improve the effect of immunotherapy. Therefore, tumor vascular normalization and immune reprogramming have a synergistic effect on each other, and can enter a benign cycle of mutual enhancement by improving TME (11, 12).

Many clinical studies explored the role of immune checkpoint inhibitors (ICIs) combined with targeted anti-angiogenic drugs, based on molecular theory basis in advanced HCC, including atezolizumab plus bevacizumab (13, 14), pembrolizumab plus lenvatinib (15), sintilimab plus bevacizumab biosimilar (16), camrelizumab plus apatinib (9). These combinations further prolonged survival of patients compared to targeted drugs. Sintilimab-bevacizumab biosimilar and camrelizumab-apatinib are approved for first-line treatment of HCC in 2021 and 2023. Sintilimab and camrelizumab are cheaper and highly available compared to atezolizumab and pembrolizumab, because they are produced domestically and favored by health insurance policies in China. So sintilimab and camrelizumab are used widely in clinical practice. The ICIs are more commonly combined with oral targeted drugs than with bevacizumab and its biosimilars, because of their economics and convenience of drug administration. So sintilimab in combination with lenvatinib and other oral targeted drugs is widely used in real-world, and the same goes for camrelizumab (17, 18).

However, the head-to-head comparisons between sintilimab and camrelizumab are few (19). We aimed to evaluate the effectiveness and safety of sintilimab versus camrelizumab, both plus targeted drugs, for advanced HCC in the real world. Subsequently, the effectiveness was compared between sintilimab-lenvatinib and camrelizumab-apatinib in first-line treatment of advanced HCC.

## 2 Materials and methods

### 2.1 Study design and patients

In this retrospective study, the medical records and imaging data of HCC patients were obtained from the a large 3A hospital in China between January 2017 and December 2023. Our study was approved by the Ethics committee of Henan Provincial People’s Hospital and owing to the retrospective nature of the study, individual consent for this analysis was waived.

All patients were diagnosed based on typical imaging features and/or biopsy or previous surgical resection (20). Patients, concurrently treated with targeted drugs and sintilimab or camrelizumab, were included in this study. The inclusion criteria are as follows: (i) age ≥18 years; (ii) Eastern Cooperative Oncology Group’s performance status (ECOG PS) score of 0-2; (iii) ChildPugh class A or B liver function; (iv) at least two usage records of ICIs and targeted drugs. The key exclusion criteria included: (i) prior diagnosis or treatment of other malignancies, severe dyspnea, cardiovascular or kidney disease, pregnant or lactating women; (ii) deficiency in imaging assessment; (iii) adjuvant or neoadjuvant therapy.

### 2.2 Treatment protocol

Sintilimab and camrelizumab were prescribed at a fixed dose of 200mg every 3 weeks. Bevacizumab biosimilar was prescribed 15mg/kg every 3 weeks by intravenous infusion (7), and six molecularly targeted drugs were administered orally. Sorafenib was administered at a dose of 400 mg twice daily, but it can be reduced to 400mg/day if the patient cannot tolerate (6). The dose of lenvatinib was dependent on weight of patients (≥60kg, 12 mg; <60kg, 8mg) (8). Regorafenib was prescribed 160mg/day during weeks 1–3 of each 4 week-cycle (21). Apatinib was prescribed 250mg/day (9). Anlotinib was administered 12mg/day during two weeks ago of each 3 week-cycle (22). Donafenib was prescribed 200mg twice daily (23).

### 2.3 Data collection and follow-up

The baseline data of patients, including patient age, sex, height, weight, accompanying disease, behavior of smoking and drinking, hepatitis B virus (HBV) or hepatitis C virus (HCV) infection, tumor stage, laboratory data, surgical situation, imaging data, and medication information were collected and gathered from patients’ medical records. Patients were followed up and monitored via phone.

### 2.4 Outcome and safety assessment

All patients were evaluated according to the modified Response Evaluation Criteria in Solid Tumors (mRECIST) (24). Progression-free survival (PFS) was defined as the time from treatment initiation of sintilimab or camrelizumab to disease progression or death from any cause. Overall survival (OS) was defined as the interval between the initial treatment and the time of death from any cause. The subgroups analysis of PFS and OS based on baseline characteristics was made to test for heterogeneity of data. Univariate and multivariate COX analysis were also done to correct for confounding factors. Then subgroup analysis of sintilimab-lenvatinib versus camrelizumab-apatinib for first-line treatment of advanced HCC was conducted.

Safety assessments and grading were recorded by the investigators from clinical examination, laboratory test results, medical records and follow-up information, using the Common Terminology Criteria for Adverse Events 5.0 (CTCAE 5.0) (25) and the management of immunotherapy-related toxicity (26) published by National Comprehensive Cancer Network (NCCN) as references.

### 2.5 Statistical analysis

Statistical analysis was performed using SPSS version 23.0 (IBM Corp., NY, USA) and the R software version 4.4.2 (http://www.r-project.org/). Differences in the clinical characteristics between the two groups were determined by chi-square test for categorical variables and independent samples t-test for continuous data. The PFS and OS were estimated using the Kaplan-Meier method, and the comparisons were analyzed using the log-rank test. Prognostic values were estimated using hazard ratios (HRs) with 95% confidence intervals (CIs). For univariate and multivariate analyses, a Cox proportional hazards regression model was constructed. The multivariate analysis included variables considered statistically significant by univariate analysis (p<0.1) and variables considered clinically closely related to the dependent variables. Mann-Whitney U test was used for ranked ordinal data, when AEs between the two groups were analyzed. Two-tailed p values < 0.05 were considered statistically significant.

## 3 Results

### 3.1 Demographics and clinical characteristics

**Figure 1:**
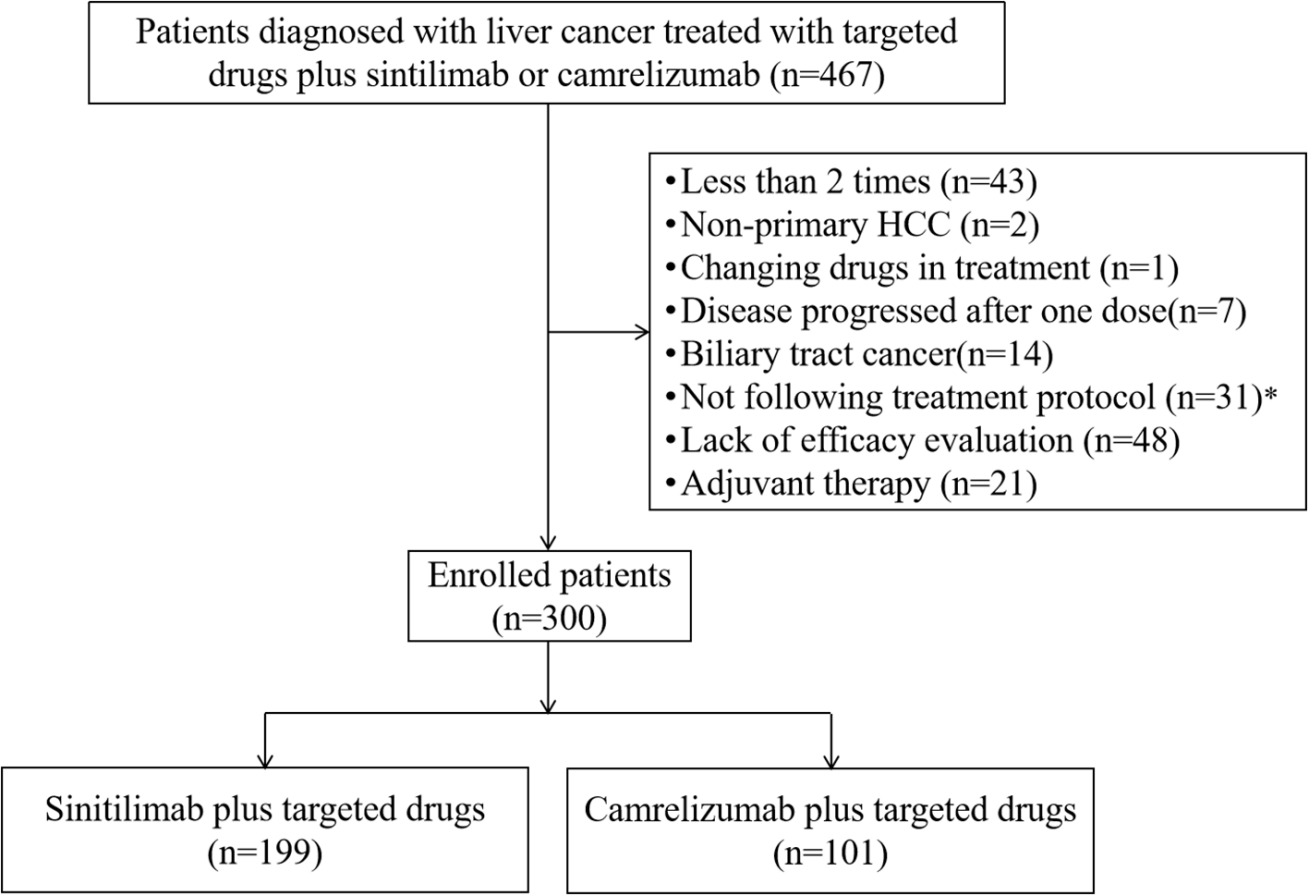
Patients’ selection flow. HCC, hepatocellular carcinoma.*Including patients have dose reductions not following the treatment protocol.

A total of 467 patients with HCC were screened, and 167 patients were excluded based on inclusion and exclusion criteria (**Figure 1**). Then, a total of 300 eligible HCC patients who were treated with targeted drugs plus sintilimab (n=199) or camrelizumab (n=101) were included in our study.

Characteristics including sex, age, body surface area, smoking and drinking history, diabetes, hypertension, HBV and HCV infection, alpha-fetoprotein (AFP) level, treatment lines, tumour metastasis, transarterial chemoembolization (TACE), radiofrequency ablation (RFA) and kinds of targeted drugs are shown in **Table 1**. There were no significant differences in the baseline characteristics between the two groups (p>0.05), excepting for the proportion of abdominal cavity metastasis (p=0.021) and targeted drugs (p=0.000).

**Table 1:** Demographics and clinical characteristics in sintilimab and camrelizumab groups.

Variables	Sintilimab (n=199)	Camrelizumab (n=101)	p
Median age (years)	55 ± 10	56 ± 11	0.211
Gender			
Male	161 (80.9%)	82 (81.2%)	0.953
Female	38 (19.1%)	19 (18.8%)	
Body surface area (m^2^)	1.77 (1.38, 2.40)	1.79 (1.43, 2.13)	0.411
Hypertension			0.480
Yes	22 (11.1%)	14(13.9%)	
No	177 (88.9%)	87 (86.1%)	
Diabetes			0.896
Yes	13 (6.5%)	7 (6.9%)	
No	186 (93.5%)	94 (93.1%)	
Smoking			0.770
Yes	106 (53.3%)	52 (51.5%)	
No	93 (46.7%)	49 (48.5%)	
Drinking			0.980
Yes	112 (56.3%)	57 (56.4%)	
No	87 (43.7%)	44 (43.6%)	
HBV infection			0.724
Yes	148 (74.4%)	77 (76.2%)	
No	51 (25.6%)	24 (23.8%)	
HCV infection			0.842
Yes	13 (6.5%)	6 (5.9%)	
No	186 (93.5%)	95 (94.1%)	
AFP level			0.196
<400 ng/ml	137 (68.8%)	62 (61.4%)	
≥ 400 ng/ml	62 (31.2%)	39 (38.6%)	
Treatment lines			0.480
1	163 (81.9%)	86 (85.1%)	
≥2	36 (18.1%)	15 (14.9%)	
Metastatic Sites			
Lymph Node	29 (14.6%)	14 (13.9%)	0.868
Lung	36 (18.1%)	18 (17.8%)	0.954
Abdominal Cavity	8 (4.0%)	11 (10.9%)	0.021
Bone	13 (6.5%)	4 (4.0%)	0.362
Others	12 (6.0%)	6 (5.9%)	0.975
TACE			0.073
Yes	136 (68.3%)	79 (78.2%)	
No	63 (31.7%)	22 (21.8%)	
RFA			
Yes	174 (87.4%)	85 (84.2%)	0.435
No	25 (12.6%)	16 (15.8%)	
Targeted drugs			0.000
Lenvatinib	160 (80.4%)	39 (38.6%)	
Apatinib	13 (6.5%)	46 (45.5%)	
Anlotinib	4 (2.0%)	2 (2.0%)	
Bevacizumab	5 (2.5%)	0	
Donafenil	4 (2.0%)	2 (2.0%)	
Regorafenib	5 (2.5%)	8 (7.9%)	
Sorafenib	8 (4.0%)	4 (4.0%)	

HBV, hepatitis B virus; HCV, hepatitis C virus; AFP, alpha-fetoprotein; TACE, transarterial chemoembolization; RFA, radiofrequency ablation.

### 3.2 Clinical outcomes

**Figure 2:**
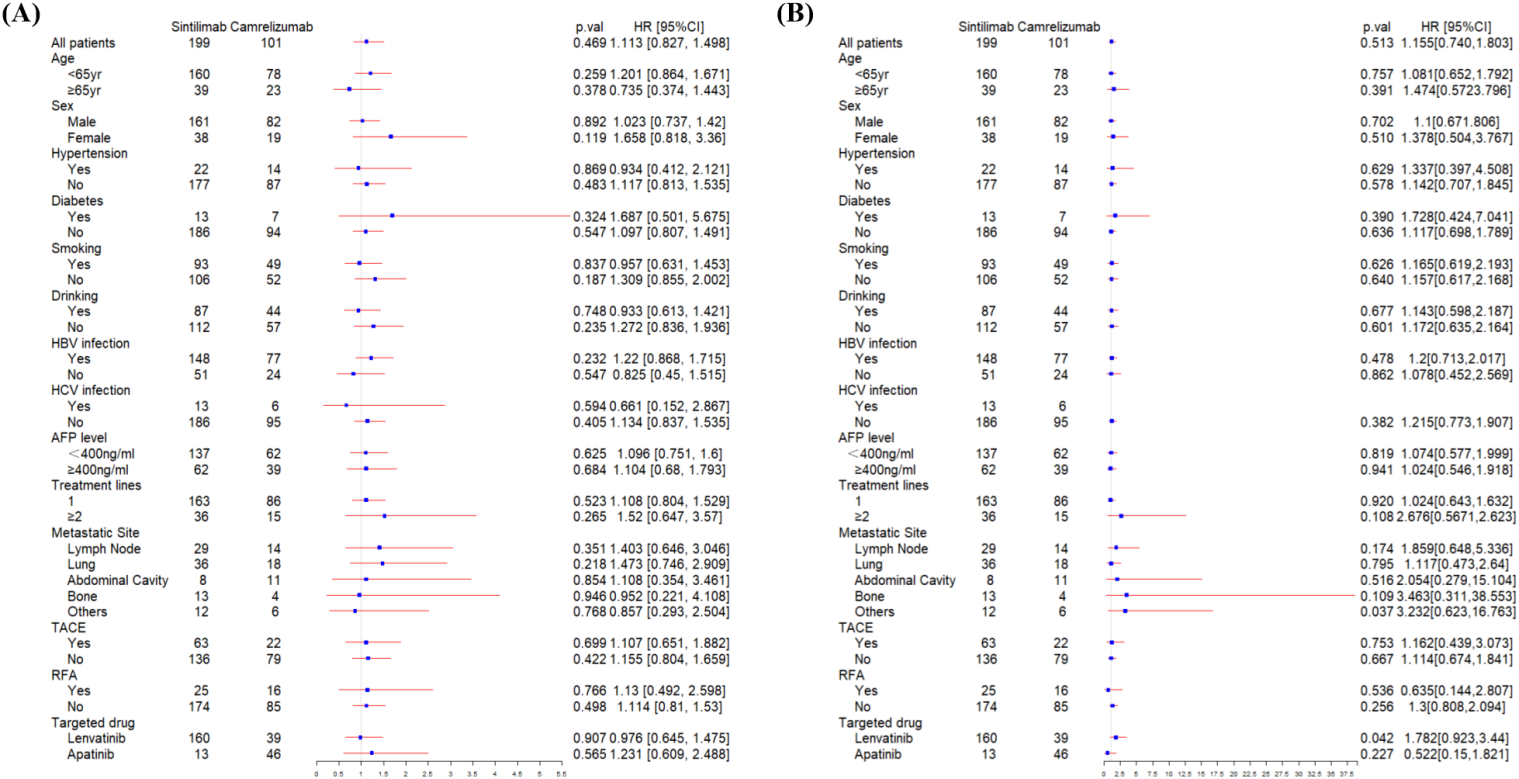
Kaplan-Meier plots of progression-free survival **(A)** and overall survival **(B)** in sintilimab and camrelizumab groups. ICIs, immune checkpoint inhibitor.

**Figure 3:**
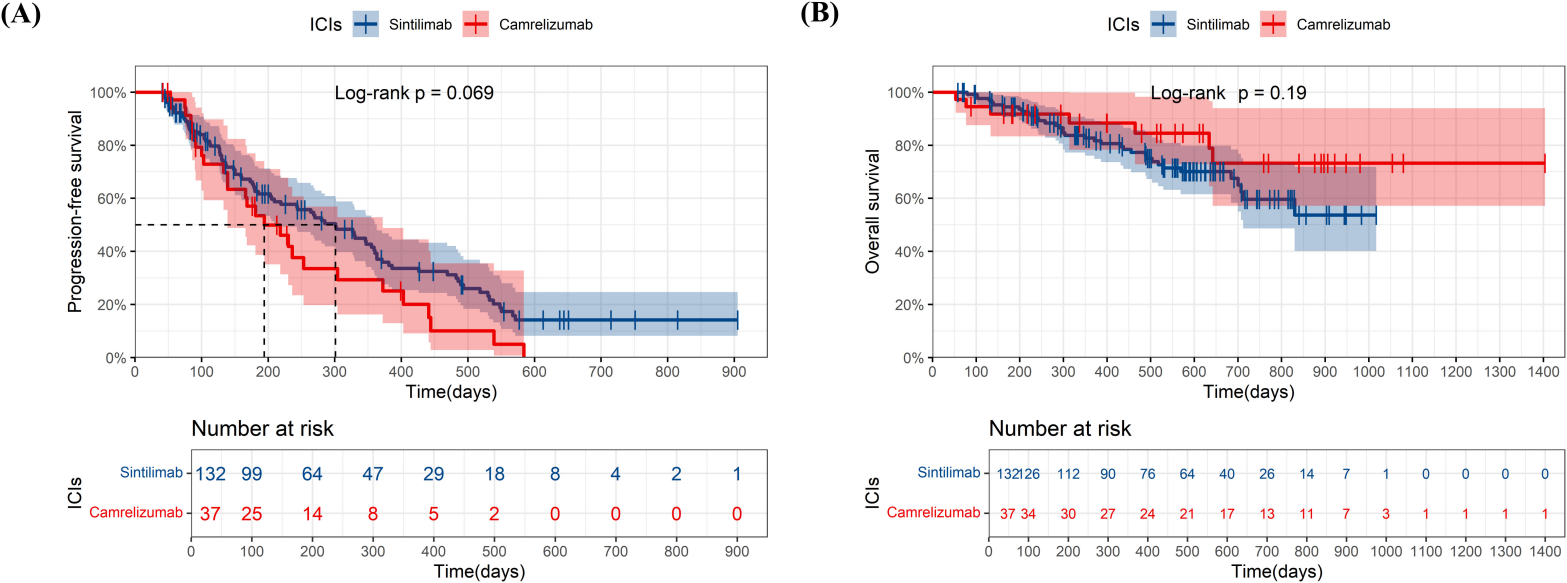
Subgroup analysis of progression-free survival **(A)** and overall survival **(B)** according to baseline characteristics. HBV, hepatitis B virus; HCV, hepatitis C virus; AFP, alpha-fetoprotein; TACE, transarterial chemoembolization; RFA, radiofrequency ablation.

At the data cutoff for the primary analysis of PFS, the median follow-up was 178 days and 156 days in sintilimab and camrelizumab groups, respectively. 131 (66%) of 199 patients in the sintilimab group and 69 (68%) of 101 patients in camrelizumab group had disease progression as assessed by mRECIST or had died. The median PFS (mPFS) in the sintilimab and camrelizumab groups was 262 days and 220 days, respectively, with no significant difference (p=0.47, **Figure 2A**). The effect of sintilimab and camrelizumab on PFS was consistent across subgroups based on baseline characteristics (**Figure 3A**).

At the date of cutoff, the median follow-up of OS was 493 days and 423 days in sintilimab and camrelizumab groups, respectively. 55 (38%) of 199 patients in the sintilimab group and 32 (31%) of 101 patients in the camrelizumab group had died. There was no statistically significant difference in OS between the two groups (p=0.51, **Figure 2B**), and neither group has reached the median OS (mOS). The effect of sintilimab and camrelizumab on OS was consistent across subgroups except for other metastatic sites and lenvatinib (**Figure 3B**). Patients with other metastatic sites [HR (95% CI): 3.232 (0.623,16.763), p=0.037] or with lenvatinib administration [HR (95% CI): 1.782 (0.923,3.44), p=0.042] in camrelizumab group had higher risk of death, compared to sintilimab group.

The univariate and multivariate COX analysis results of the factors on PFS and OS are shown in **Supplementary Material Tables S1**, **S2**. Multivariable Cox analysis identified the number of metastatic sites (≥2 vs. 0-1; HR=1.823, 95% CI: 1.162-2.862, p=0.009) and AFP level ≥400ng/mL (HR=1.539, 95% CI: 1.113-2.127, p=0.009) as independent predictors of shorter PFS. Similar associations were observed for OS (metastatic sites: HR=2.272, 95% CI: 1.238-4.172, p=0.008; AFP: HR=2.775, 95% CI: 1.768-4.355, p=0.000). Diabetes was related to shorter PFS (HR=1.975, 95% CI: 1.12-3.485, p=0.019). HCV infection and RFA were separately independent predictors of longer PFS and OS (HCV infection for PFS: HR=0.409, 95% CI: 0.186-0.901, p=0.027; RFA for OS: HR=0.381, 95% CI: 0.171-0.85, p=0.018). But our primary outcomes were not affected by these factors, despite incomplete matching of baseline levels (PFS: HR=0.918, 95% CI: 0.653-1.291, p=0.623; OS: HR=1.106, 95% CI: 0.66-1.851, p=0.702).

Propensity score matching (PSM) was conducted to eliminate the effect of baseline characteristics (abdominal cavity metastasis and targeted drugs) with caliper for 0.1, and results were showed in **Supplementary Material Figure S1**. After adjusting for confounding factors by PSM, no statistically significant difference was still found between sintilimab and camrelizumab groups in the comparison of the PFS (mPFS: 280 vs. 247 days, p=0.95) and OS (p=0.94), suggesting that confounding factors (abdominal cavity metastasis and targeted drugs) have a small effect on the primary outcomes.

### 3.3 Subgroup analysis of sintilimab-lenvatinib versus camrelizumab-apatinib

**Figure 4:**
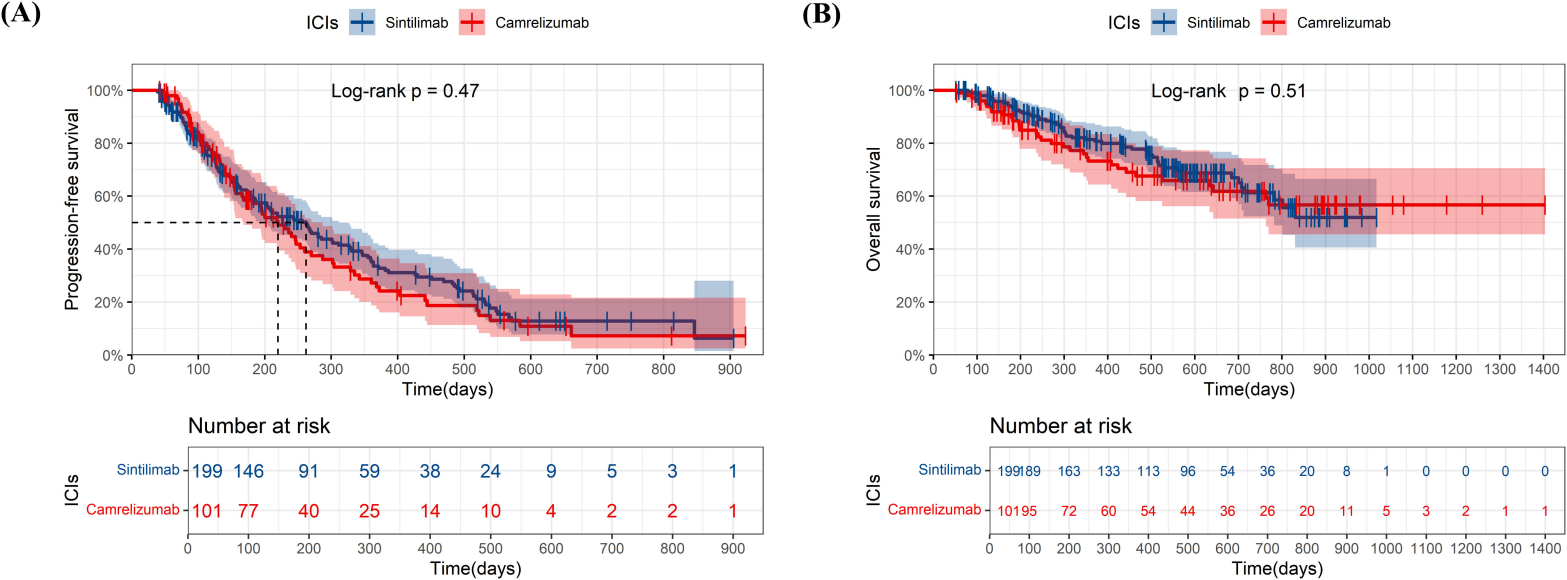
Kaplan-Meier plots of progression-free survival **(A)** and overall survival **(B)** in sintilimab plus lenvatinib group and camrelizumab plus apatinib group. ICIs, immune checkpoint inhibitor.

We compare the effectiveness between sintilimab-lenvatinib and camrelizumab-apatinib for first-line treatment (**Figures 4A, B**). No significant differences in PFS (p=0.069) and OS (p=0.19) were observed between two combinations, although combination of the sintilimab-lenvatinib had a longer mPFS than the camrelizumab-apatinib (301 days versus 194 days). Neither group reached the mOS.

### 3.4 Safety

192 (96.5%) of 199 patients and 98 (97.0%) of 101 patients experienced at least one adverse event (AE) of any grade in sintilimab and camrelizumab groups (**Table 2**). The numbers of patients discontinued due to AEs in sintilimab and camrelizumab groups were 3 (1.5%) and 2 (2.0%), respectively. The number of patients with dose adjustments according to treatment protocol was 4 (2%) in sintilimab group and 1 (1%) in camrelizumab group. The incidences of serious adverse events (SAE), defined as AE of grade 3-4, were 41.2% and 52.5%, respectively. There were no significant differences in AEs whether it’s grade 1–2 or grade3–4 between two groups (p=0.071). The main AEs of any grade in two groups included decreased platelet count, decreased white blood cell, decreased neutrophil count, decreased hemoglobin, increased alanine aminotransferase, increased aspartate aminotransferase, increased blood bilirubin and hyponatremia, et al.

**Table 2:** Summary of adverse events in sintilimab and camrelizumab groups.

Items	Sintilimab (n=199)		Camrelizumab (n=101)		p
	Grade 1-2	Grade 3-4	Grade 1-2	Grade 3-4	
Treatment-emergent adverse events	110 (55.3%)	82 (41.2%)	45 (44.6%)	53 (52.5%)	0.071
Decreased white blood cell	84 (42.2%)	24 (12.1%)	44 (43.6%)	11 (10.9%)	0.941
Decreased neutrophil count	79 (39.7%)	32 (16.1%)	38 (37.6%)	21 (20.8%)	0.458
Decreased platelet count	103 (51.8%)	42 (21.1%)	46 (45.5%)	21 (20.8%)	0.407
Decreased hemoglobin	75 (37.7%)	5 (2.5%)	43 (42.6%)	1 (1%)	0.660
Increased alanine aminotransferase	52 (26.1%)	9 (4.5%)	30 (29.7%)	6 (5.9%)	0.369
Increased aspartate aminotransferase	84 (42.2%)	11 (5.5%)	46 (45.5%)	11 (10.9%)	0.088
Increased blood bilirubin	65 (32.7%)	14 (7%)	36 (35.6%)	12 (11.9%)	0.138
Increased gamma-glutamyl transferase	50 (25.1%)	6 (3%)	23 (22.8%)	4 (4%)	0.842
Proteinuria	46 (23.1%)	3 (1.5%)	26 (25.7%)	1 (1%)	0.715
Increased creatinine	11 (5.5%)	0	8 (7.9%)	0	0.422
hypokalemia	55 (27.6%)	12 (6%)	24 (23.8%)	10 (9.9%)	0.825
Hyponatremia	65 (32.7%)	8 (4%)	39 (38.6%)	4 (4%)	0.350
Increased blood thyroid-stimulating hormone	12 (6%)	12 (6%)	10 (9.9%)	6 (5.9%)	0.396
Asthenia	16 (8%)	1 (0.5%)	6 (5.9%)	0	0.419
Appetite decreased	21 (10.6%)	0	14 (13.9%)	0	0.400
Abdominal pain	7 (3.5%)	0	3 (3%)	0	0.803
Abdominal distension	10 (5%)	0	7 (6.9%)	0	0.501
Diarrhea	12 (6%)	0	3 (3%)	0	0.251
Nausea	12 (6%)	0	6 (5.9%)	0	0.975
Vomiting	11 (5.5%)	0	3 (3)	0	0.322
Palmar-plantar erythrodysaesthesia syndrome	4 (2%)	1 (0.5%)	0 (0)	1 (1%)	0.381
Rash	16 (8%)	2 (1%)	1 (1%)	4 (4%)	0.245
Elevated blood pressure	1 (0.5%)	1 (0.5%)	0 (0)	1 (1%)	0.993
Blurred vision	2 (1%)	0	1 (1%)	0	0.990
Oral mucositis	1 (0.5%)	1 (0.5%)	1 (1%)	0	0.987
Hoarseness	2 (1%)	0	0	0	0.313

## 4 Discussion

Our study revealed a real-world comparison of the effectiveness and safety between sintilimab and camrelizumab for the treatment of advanced HCC. There was no statistically significant difference in PFS and OS between sintilimab and camrelizumab groups. And there was no significant difference in AEs between two groups. In subgroup analysis, no significant difference was observed in PFS and OS between sintilimab-lenvatinib and camrelizumab-apatinib for first-line treatment, although combination of the sintilimab-lenvatinib had a longer mPFS than the camrelizumab group.

Sintilimab and camrelizumab are both monoclonal antibodies that target the PD-1 receptor on T cells and have been approved for the treatment of advanced HCC in China. A retrospective study (19) on effectiveness and safety of toripalimab (n=23), camrelizumab (n=33), and sintilimab (n=14) in HCC patients with HBV showed that no significant difference in PFS and OS among them was observed. A network meta-analysis, about first-line systemic treatment for HCC, showed that camrelizumab plus apatinib showed a statistically significant superiority in all comparisons, such as sintilimab plus bevacizumab (27). Another network meta-analysis (28), about phase III trials of first-line systemic therapy for advanced HCC, revealed sintilimab plus bevacizumab biosimilars and camrelizumab plus apatinib reduced the risk of death with HRs of 0.57 and 0.62 compared with sorafenib indicating a slight advantage of sintilimab. Therefore, it’s ambiguous as to who is more effective between sintilimab and camrelizumab, and the evidence for direct comparisons is insufficient. Our study shows that sintilimab and camrelizumab have equal clinical effectiveness in all patients, but sintilimab-lenvatinib is possibly superior to camrelizumab-apatinib in first-line treatment with HCC.

The randomized controlled trial (RCT) study (ORIENT-32) (16) showed that patients in the sintilimab-bevacizumab biosimilar group had a significantly longer mPFS (4.6 months) than did patients in the sorafenib group. A real-world analysis of sintilimab-bevacizumab biosimilar in patients with advanced HCC indicated that mPFS was 238 days (29). The mPFS of sintilimab plus anlotinib as first-line treatment for advanced HCC was 12.2 months in real-world study (30). So the survival time of sintilimab plus targeted drugs was different between RCT and real-world studies, and was inconsistent among real-world studies also. The same can be said for camrelizumab. The mPFS was 5.6 months in camrelizumab-apatinib group as first-line therapy for unresectable HCC in the RCT study (CARES-310) (9). The mPFS was 9.6 months in camrelizumab and apatinib group (31). The mPFS in second-line treatment of camrelizumab combined with apatinib was 10.5 months (32). The mPFS was 10.3 months in treatment group of camrelizumab and lenvatinib as first-line treatment of unresectable HCC (33). The mPFS of camrelizumab plus apatinib in first-line and second-line setting cohorts was 5.7 months and 5.5 months respectively in a phase 2 trial (34). In our study, the mPFS of sintilimab and camrelizumab is 262 days and 220 days in all patients, and 301 days and 194 days in patients receiving first-line treatment, respectively. The differences in survival time between researches are possibly due to the usage and dosage of ICIs and targeted drugs, patients included, follow-up time and so on.

The distribution of abdominal cavity metastasis is unequal between sintilimab and camrelizumab groups. But it doesn’t affect our primary outcomes. Because the subgroups analysis of PFS and OS based on baseline characteristics was consistent with primary outcomes, and results of the survival analysis after PSM were consistent with the primary outcomes, too. The distributional difference in type of targeted drugs was related to physicians’ drug habits, drug instructions, medical insurance policy and drug availability in hospital. So we performed survival analysis between two common combinations (sintilimab-lenvatinib and camrelizumab-apatinib) for first-line treatment of HCC. No significant difference was seen between the two groups in terms of PFS and OS, despite an advantage in the sintilimab group in terms of mPFS. However, this result is still questionable: (i) the sample size in the camrelizumab group is small (n=37); (ii) it was unknown whether the baseline data of the two groups were matched, as this was only a subgroup analysis. Therefore, future confirmation is needed in large-sample and head-to-head RCTs or real-world studies.

The difference in other metastatic sites, which was observed in subgroup analysis of OS, is not clinically significant in our opinion, because the sample size is small (sintilimab 12 vs camrelizumab 6). Sintilimab-lenvatinib showed longer OS then camrelizumab-lenvatinib showing the advantage of sintilimab than camrelizumab, which maybe fits with subgroup analysis of sintilimab-lenvatinib versus camrelizumab-apatinib.

Sintilimab and camrelizumab had an acceptable safety profile. No new AE was reported in the study. AEs in laboratory test results were collected easily from electronic medical record. But other AEs, such as reactive cutaneous capillary endothelial proliferation (RCCEP), nausea and vomiting, were difficult to obtained, because they were extracted from patient’s medical records or telephone follow-up. These AEs would be failed to be obtained if they were not recorded in medical records by doctors or patient was unclear or had forgotten. So they were possibly not captured in some patients, leading to low incidence in our study.

Comparisons between sintilimab and camrelizumab for the treatment of advanced HCC have some limitations. Firstly, residual confounding may persist due to imbalanced baseline characteristics, such as the higher proportion of abdominal cavity metastasis in the camrelizumab group and preferential use of lenvatinib/apatinib. These imbalances reflect real-world clinical practice, where treatment selection is often influenced by factors like medical insurance policy or drug availability. Future randomized trials with protocol-defined combinations are warranted to validate our findings. Secondly, the median duration of follow-up was not long enough and the mOS, as a major primary endpoint, was not met. Thirdly, the analyzable data were limited to those available in electronic medical record and telephone follow-up, so that certain AEs, such RCCEP, nausea and vomiting might be underreported. Fourthly, this is a single-center retrospective study. Therefore, we hope that multi-center, head-to-head clinical studies, including RCTs and real-world studies which can complement each other, will validate our results.

In conclusion, our study shows that sintilimab and camrelizumab, both plus targeted drugs, have equal clinical effectiveness and incidence of AEs. There is no difference between sintilimab-lenvatinib and camrelizumab-apatinib in first-line treatment for advanced HCC, since the former is seemed to be a slight superiority.

## Data Availability

The original contributions presented in the study are included in the article/**Supplementary Material**. Further inquiries can be directed to the corresponding author.
