# Supplementary figures and images for: The safety and effectiveness of sintilimab versus camrelizumab, both plus targeted drugs, in advanced hepatocellular carcinoma
Source: Front Immunol. 2025 Jun 23;16:1585956. doi: 10.3389/fimmu.2025.1585956 (PMC12230080; doi:10.3389/fimmu.2025.1585956)

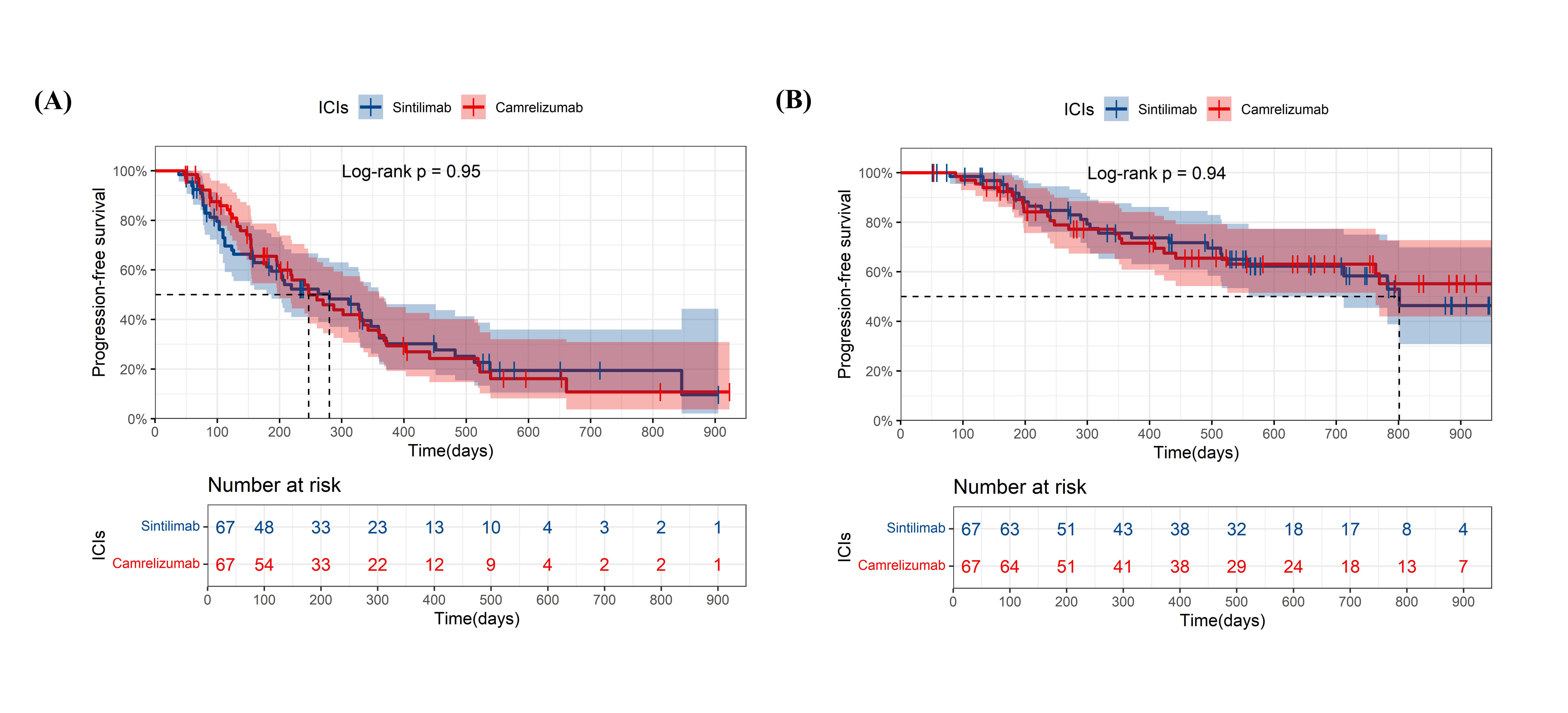

Supplement: Supplementary file 1 [file Image1.tif]
