# Supplementary material for: The safety and effectiveness of sintilimab versus camrelizumab, both plus targeted drugs, in advanced hepatocellular carcinoma
Source: Front Immunol. 2025 Jun 23;16:1585956. doi: 10.3389/fimmu.2025.1585956 (PMC12230080; doi:10.3389/fimmu.2025.1585956)
Supplement: Supplementary file 2 [file Table1.docx]

Supplementary Material

Table S1 The univariate and multivariate Cox regression analysis of PFS

| Baseline characteristics | Univariate analysis | | Multivariate analysis* | |
| --- | --- | --- | --- | --- |
|  | HR (95% CI) | P*‑*value | HR (95% CI) | P*‑*value |
| Age (＞65 vs. ≤65years) | 1.012(0.705-1.452) | 0.949 |  |  |
| Gender (female vs. male) | 0.92(0.646-1.31) | 0.643 |  |  |
| Hypertension | 1.061(0.692-1.627) | 0.787 |  |  |
| Diabetes | 1.643(0.949-2.846) | 0.076 | 1.975(1.12-3.485) | 0.019 |
| Smoking | 1.099(0.833-1.451) | 0.504 |  |  |
| Drinking | 1.292(0.978-1.707) | 0.072 | 1.297(0.973-1.729) | 0.076 |
| HBV infection | 1.141(0.821-1.587) | 0.433 | 0.85(0.584-1.237) | 0.395 |
| HCV infection | 0.448(0.22-0.91) | 0.026 | 0.409(0.186-0.901) | 0.027 |
| AFP level (≥ 400 vs. <400 ng/ml) | 1.417(1.055-1.903) | 0.021 | 1.539(1.113-2.127) | 0.009 |
| Treatment lines (≥2 vs. 1) | 1.47(1.01-2.14) | 0.044 | 1.386(0.937-2.049) | 0.102 |
| Metastatic Sites |  |  |  |  |
| Non | Reference |  | Reference |  |
| 1 | 1.173(0.851-1.616) | 0.33 | 1.197(0.859-1.667) | 0.288 |
| ≥2 | 1.963(1.279-3.014) | 0.002 | 1.823(1.162-2.862) | 0.009 |
| TACE | 1.164(0.87-1.557) | 0.308 | 1.26(0.918-1.728) | 0.153 |
| RFA | 0.621(0.407-0.946) | 0.027 | 0.641(0.406-1.012) | 0.056 |
| Targeted drugs |  |  |  |  |
| Others | Reference |  | Reference |  |
| Lenvatinib | 0.838(0.55-1.277) | 0.41 | 0.711(0.455-1.112) | 0.135 |
| Apatinib | 1.181(0.72-1.938 | 0.51 | 1.131(0.662-1.933) | 0.653 |
| Group (camrelizumab vs. sintilimab) | 1.114(0.832-1.492) | 0.470 | 0.918(0.653-1.291) | 0.623 |

PFS, progression-free survival; HBV, hepatitis B virus; HCV, hepatitis C virus; AFP, alpha-fetoprotein; TACE, transarterial chemoembolization; RFA, radiofrequency ablation. *Variables that met one of the following criteria were included in the multivariate analysis: (ⅰ) Variables considered statistically significant by univariate analysis (p<0.1); (ⅱ) Variables considered clinically closely related to the dependent variable.

Table S2 The univariate and multivariate Cox regression analysis of OS

| Baseline characteristics | Univariate analysis | | Multivariate analysis* | |
| --- | --- | --- | --- | --- |
|  | HR (95% CI) | P*‑*value | HR (95% CI) | P*‑*value |
| Age (＞65 vs. ≤65years) | 1.443(0.872-2.387) | 0.154 |  |  |
| Gender (female vs. male) | 1.068(0.629-1.815) | 0.807 |  |  |
| Hypertension | 0.917(0.487-1.728) | 0.79 |  |  |
| Diabetes | 1.683(0.841-3.368) | 0.141 |  |  |
| Smoking | 1.091(0.716-1.66) | 0.686 |  |  |
| Drinking | 1.171(0.768-1.784) | 0.464 |  |  |
| HBV infection | 0.956(0.594-1.54) | 0.854 | 0.705(0.424-1.174) | 0.179 |
| HCV infection | 0.294(0.072-1.195) | 0.087 | 0.257(0.059-1.122) | 0.071 |
| AFP level (≥ 400 vs. <400 ng/ml) | 2.486(1.627-3.796) | 0 | 2.775(1.768-4.355) | 0.000 |
| Treatment lines (≥2 vs. 1) | 0.768(0.397-1.486) | 0.434 | 0.828(0.419-1.635) | 0.587 |
| Metastatic Sites |  |  |  |  |
| Non |  |  |  |  |
| 1 | 1.239(0.758-2.025) | 0.393 | 1.087(0.659-1.792) | 0.745 |
| ≥2 | 2.619(1.461-4.695) | 0.001 | 2.272(1.238-4.172) | 0.008 |
| TACE | 0.77(0.475-1.249) | 0.29 | 0.754(0.459-1.238) | 0.264 |
| RFA | 0.356(0.164-0.773) | 0.009 | 0.381(0.171-0.85) | 0.018 |
| Targeted drugs |  |  |  |  |
| Others |  |  |  |  |
| Lenvatinib | 0.908(0.506-1.63) | 0.747 | 0.773(0.424-1.408) | 0.400 |
| Apatinib | 0.778(0.375-1.612) | 0.5 | 0.639(0.289-1.414) | 0.269 |
| Group (camrelizumab vs. sintilimab) | 1.157(0.747-1.794) | 0.514 | 1.106(0.66-1.851) | 0.702 |

OS, overall survival; HBV, hepatitis B virus; HCV, hepatitis C virus; AFP, alpha-fetoprotein; TACE, transarterial chemoembolization; RFA, radiofrequency ablation. *Variables that met one of the following criteria were included in the multivariate analysis: (ⅰ) Variables considered statistically significant by univariate analysis (p<0.1); (ⅱ) Variables considered clinically closely related to the dependent variable.

Figure S1. Kaplan-Meier plots of progression-free survival **(A)** and overall survival **(B)** in sintilimab and camrelizumab groups after propensity score matching. ICIs, immune checkpoint inhibitor.
